# 1.65 Å resolution structure of the AraC-family transcriptional activator ToxT from *Vibrio cholerae*

**DOI:** 10.1107/S2053230X1601298X

**Published:** 2016-08-26

**Authors:** Jiaqin Li, Graham Wehmeyer, Scott Lovell, Kevin P. Battaile, Susan M. Egan

**Affiliations:** a Department of Molecular Bioscience, The University of Kansas, 8031 Haworth, 1200 Sunnyside Avenue, Lawrence, KS 66045, USA; b Protein Structure Laboratory, Shankel Structural Biology Center, The University of Kansas, 2034 Becker Drive, Lawrence, KS 66047, USA; c IMCA-CAT, Hauptman–Woodward Medical Research Institute, 9700 South Cass Avenue, Building 435A, Argonne, IL 60439, USA

**Keywords:** AraC, ToxT, *Vibrio cholerae*, crystal structure, palmitoleic acid, pathogenesis

## Abstract

ToxT is an AraC-family transcriptional activator protein that controls the expression of key virulence factors in *Vibrio cholerae*, the causative agent of cholera. ToxT directly activates the expression of the genes that encode the toxin-coregulated pilus and cholera toxin, and also positively auto-regulates its own expression from the *tcp* promoter. The crystal structure of ToxT has previously been solved at 1.9 Å resolution (PDB entry 3gbg). In this study, a crystal structure of ToxT at 1.65 Å resolution with a similar overall structure to the previously determined structure is reported. However, there are distinct differences between the two structures, particularly in the region that extends from Asp101 to Glu110. This region, which can influence ToxT activity but was disordered in the previous structure, can be traced entirely in the current structure.

## Abstract 2

A crystal structure of ToxT at 1.65 Å resolution with a similar overall structure to the previously determined structure is reported. A region that extends from Asp101 to Glu110, which can influence ToxT activity but was disordered in the previous structure, can be traced entirely in the current structure.

## 1. Introduction   

The AraC family of transcriptional activators, with members present in over 70% of sequenced bacterial genomes, is defined by a DNA-binding domain containing two helix–turn–helix motifs (Ramos *et al.*, 1990 ▸; Gallegos *et al.*, 1993 ▸, 1997 ▸; Egan, 2002 ▸; Ibarra *et al.*, 2008 ▸). Many AraC-family proteins have a second domain, the sequence of which shares sequence similarity within subsets of the family but not the entire family (Gallegos *et al.*, 1997 ▸). The most common roles of the non-DNA-binding domain are effector binding and/or dimerization. AraC-family members typically activate the expression of genes involved in carbon metabolism, stress responses or virulence (Gallegos *et al.*, 1993 ▸, 1997 ▸; Egan, 2002 ▸; Tobes & Ramos, 2002 ▸; Ibarra *et al.*, 2008 ▸). ToxT is an AraC-family transcriptional activator of *Vibrio cholerae* virulence-gene expression, with a C-terminal DNA-binding domain and an N-terminal domain involved in dimerization and effector binding (Lowden *et al.*, 2010 ▸). ToxT directly activates the expression of the genes encoding the toxin-coregulated pilus (TCP), which is essential for colonization of the human intestine, and the cholera toxin (CT), the cause of the diarrheal disease that is characteristic of cholera (Champion *et al.*, 1997 ▸; DiRita *et al.*, 1991 ▸; Higgins *et al.*, 1992 ▸). ToxT has also been shown to positively auto-regulate its own expression from the *tcp* promoter (Brown & Taylor, 1995 ▸; Yu & DiRita, 1999 ▸). In *V. cholerae*, ToxT-dependent gene activation is inhibited by both bile and individual unsaturated fatty acids found in bile (Schuhmacher & Klose, 1999 ▸; Chatterjee *et al.*, 2007 ▸). The full-length structure of ToxT determined by Lowden *et al.* (2010 ▸) has the fatty acid *cis*-palmitoleic acid (PAM) bound to the N-terminal domain. Although oleic acid is likely to be the physiological effector of ToxT given its high concentration in bile, both PAM and oleic acid have been shown to reduce the expression of *tcp* and *ctx in vivo* and to reduce DNA binding by ToxT *in vitro* (Lowden *et al.*, 2010 ▸). Therefore, the structure obtained by Lowden *et al.* (2010 ▸) is expected to represent the non-activating state of ToxT, where its ability to bind DNA and activate transcription is reduced compared with its activating conformation without effector bound.

The previously determined 1.9 Å resolution ToxT crystal structure (PDB entry 3gbg; Lowden *et al.*, 2010 ▸) shows that ToxT has the same overall domain architecture as the predicted AraC protein: each of the ToxT monomers comprises an N-terminal effector-binding and dimerization domain that shares sequence similarity with the AraC N-terminal domain, and a C-terminal DNA-binding domain. ToxT was the first AraC-family protein from the same subset of the family as AraC to have its full-length structure resolved at high resolution. However, the structure determined by Lowden *et al.* (2010 ▸) contains a disordered region between residues Asp101 and Glu110 within the N-terminal domain. Childers *et al.* (2007 ▸) have shown that alanine substitutions at residues within the disordered region in the 3gbg structure, Met103, Arg105 and Asn106, increase the activation of the *ctxA* promotor by threefold to fourfold compared with wild-type ToxT, indicating that this region is important for proper ToxT activation (Childers *et al.*, 2007 ▸). Hung *et al.* (2005 ▸) have shown that replacing the leucine at residue 114 with a proline confers resistance to virstatin, a small-molecule inhibitor of ToxT, suggesting that the nearby disordered region may also be important for inhibition by virstatin (Hung *et al.*, 2005 ▸; Shakhnovich *et al.*, 2007 ▸). Here, we report a crystal structure of ToxT at 1.65 Å resolution (PDB entry 4mlo) in which the region spanning Asp101–Glu110 could be modeled.

## 2. Materials and methods   

### 2.1. Protein purification and crystallization   

The expression and purification of ToxT was performed as described previously (Lowden *et al.*, 2010 ▸), with a few exceptions. Briefly, ToxT was overexpressed as a ToxT–intein–chitin-binding domain fusion from plasmid pTXB1 (New England Biolabs), the same construct as used by Lowden *et al.* (2010 ▸), by autoinduction in ZYM-5052 medium (Studier, 2005 ▸) with 200 µg ml^−1^ ampicillin using *Escherichia coli* strain BL21 (DE3) (New England Biolabs). This strain differed from the BL21-CodonPlus (DE3)-RIL (Stratagene) strain used by Lowden *et al.* (2010 ▸) as we found that ToxT was highly overexpressed in the basic BL21 (DE3) strain. The initial purification was carried out using a chitin-affinity column (New England Biolabs) with gravity flow. ToxT was cleaved from the intein–chitin-binding domain by the addition of 100 m*M* dithiothreitol (DTT) to cleavage buffer (20 m*M* Tris pH 8.0, 1 m*M* EDTA, 150 m*M* NaCl) and incubation for 16 h at 4°C. ToxT was eluted from the column, and the eluent, which contained untagged ToxT, was loaded onto a HiTrap SP Sepharose Fast Flow cation-exchange column (GE Healthcare) in buffer consisting of 20 m*M* Tris–HCl pH 6.8, 33.3 m*M* DTT, 50 m*M* NaCl. ToxT was eluted using a gradient from 100% buffer *A* (0.05 *M* NaCl) to 100% buffer *B* (1 *M* NaCl), with the protein peak corresponding to ToxT eluting at 88% buffer *B*. The fractions containing purified ToxT protein were combined and then concentrated to 1.65 mg ml^−1^ for crystallization screening using an Amicon ultracentrifugal filter unit (Millipore) with a molecular-weight cutoff of 10 kDa. All crystallization screening was conducted in Compact Jr or Clover Jr (Rigaku Reagents) sitting-drop vapor-diffusion plates incubated at 293 K using 0.75 µl protein solution and 0.75 µl crystallization solution equilibrated against 75 µl of the latter. Crystals displaying needle (∼100 × 10 µm) or plate (∼60 × 20 µm) morphology formed overnight from various screens. The plate-shaped crystals which were used for data collection were obtained using condition H10 [5%(*w*/*v*) PEG 4000, 10%(*v*/*v*) 2-propanol, 0.1 *M* MES pH 6.5, 200 m*M* MgCl_2_] from the ProPlex HT screen (Molecular Dimensions), a condition that was significantly different from the crystallization condition identified by Lowden *et al.* (2010 ▸). Crystals were transferred into a fresh drop composed of 80% crystallization solution and 20% ethylene glycol and stored in liquid nitrogen.

### 2.2. Data collection and structure refinement   

X-ray diffraction data were collected on beamline 17-ID at the Advanced Photon Source using a Dectris PILATUS 6M pixel-array detector. Intensities were integrated using *XDS* (Kabsch, 1988 ▸), and Laue class analysis and data scaling were performed with *AIMLESS* (Evans & Murshudov, 2013 ▸), which suggested that the highest probability Laue class was 2/*m* with space group *P*2_1_. The Matthews coefficient (Matthews, 1968 ▸; *V*_M_ = 2.3 Å^3^ Da^−1^, 46.8% solvent content) suggested that the asymmetric unit contained a single molecule. Structure solution was conducted by molecular replacement with *Phaser* (McCoy *et al.*, 2007 ▸) *via* the *PHENIX* (Adams *et al.*, 2010 ▸) interface using a previously determined non-isomorphous structure of ToxT (PDB entry 3gbg; Lowden *et al.*, 2010 ▸) as the search model. All space groups with point symmetry 2 were tested and the top solution was obtained for a single molecule in the asymmetric unit in space group *P*2_1_. Structure refinement and manual model building were conducted with *PHENIX* and *Coot* (Emsley *et al.*, 2010 ▸), respectively. TLS refinement (Painter & Merritt, 2006 ▸; Winn *et al.*, 2001 ▸) was incorporated in the latter stages to model anisotropic atomic displacement parameters. Structure validation was conducted with *MolProbity* (Chen *et al.*, 2010 ▸) and figures were prepared using the *CCP*4*mg* package (McNicholas *et al.*, 2011 ▸). The final refinement and model statistics are given in Table 1 ▸. Refined atomic coordinates and experimental structure factors have been deposited in the Protein Data Bank (PDB entry 4mlo).

## 3. Results and discussion   

**Figure 1:**
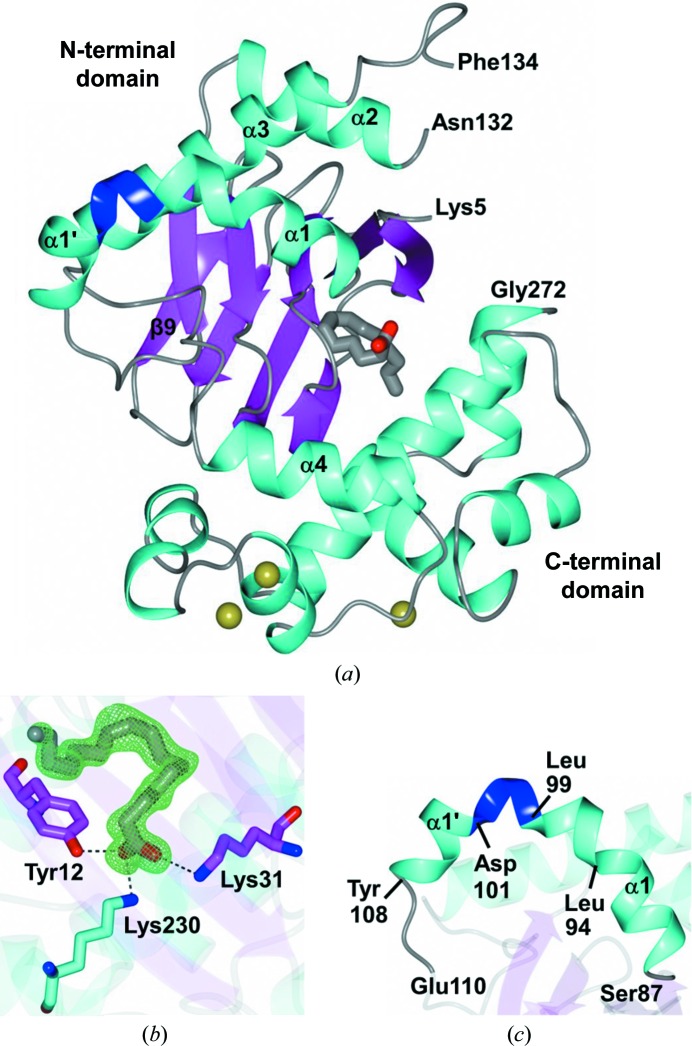
(*a*) Asymmetric unit of ToxT (PDB entry 4mlo) colored by secondary structure. The N- and C-terminal residues (Lys5 and Gly272) of the model are indicated along with the disordered region between Asn132 and Phe134. The 3_10_-helix spanning Leu99–Asp101 is colored blue. The PAM molecule and chloride ions are shown as cylinders and gold spheres, respectively. (*b*) *F*_o_ − *F*_c_ OMIT map contoured at 3σ (green mesh) for PAM and associated hydrogen bonds (dashed lines) to ToxT residues. (*c*) Enlarged view of the region from Ser87 to Glu110. Helix α1 spans Ser87–Ile98 and contains a kink at Leu94. This is followed by a 3_10_-helix spanning Leu99–Asp101 and a shorter helix from Leu102 to Leu107 referred to as α1′.

The final model of ToxT could be traced in the electron-density map from Lys5 to Gly272, except for the disordered Gly133, which is located in a loop connecting helix α2 to helix α3 (Fig. 1 ▸ *a*). Electron density consistent with PAM was also present (Fig. 1 ▸ *b*), as was observed in the original ToxT structure (PDB entry 3gbg; Lowden *et al.*, 2010 ▸), although PAM was not added in either case but was acquired from the expression host. Interestingly, Asp101–Glu110 could be modeled in this structure, which included helix α1 and a loop region that connects this helix to the β9 sheet. The helix in our structure can be thought of as containing two segments, which we refer to as α1 and α1′ to be consistent with the prior secondary-structure assignment for PDB entry 3gbg (Lowden *et al.*, 2010 ▸; Fig. 1 ▸ *c*). In addition, three chloride ions were modeled in the C-terminal region of ToxT, which were assigned based on the coordination distances (∼3.1–3.3 Å) to neighboring residues and water molecules. When water molecules were initially assigned to the chloride sites, positive electron density was observed following refinement, indicating an underestimation of electrons. Therefore, the modeling of chloride ions at these sites was consistent with the observed electron density and coordination.

**Figure 2:**
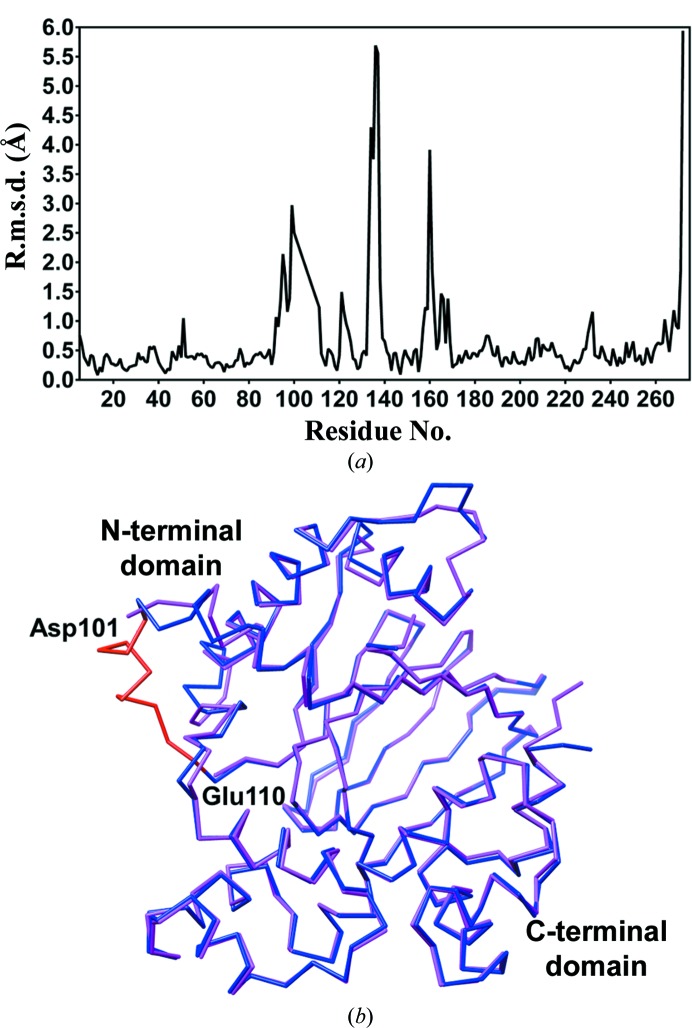
(*a*) Plot of r.m.s.d. per residue between C^α^ atoms for ToxT (PDB entry 4mlo) and the previously determined structure (PDB entry 3gbg; Lowden *et al.*, 2010 ▸). (*b*) Superposition of PDB entry 4mlo (blue) with the previously determined structure (PDB entry 3gbg; Lowden *et al.*, 2010 ▸; magenta). The previously disordered region from Asp101 to Glu110 is highlighted in red.

**Figure 3:**
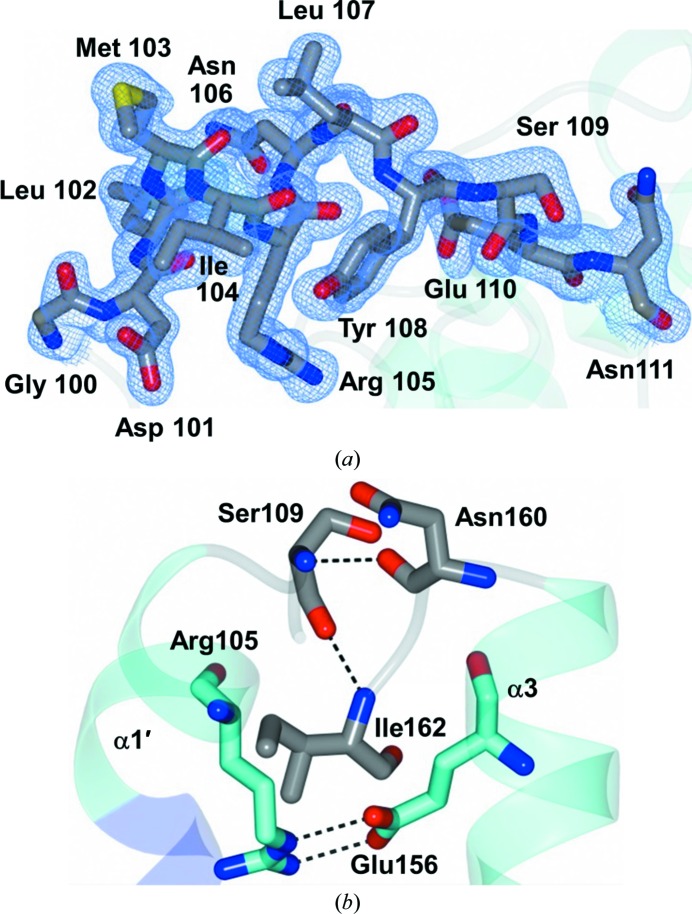
Loop region between α1′ and β9. (*a*) 2*F*_o_ − *F*_c_ map contoured at 1σ (blue mesh) for residues Gly100–Asn111 which were disordered in PDB entry 3gbg (Lowden *et al.*, 2010 ▸). (*b*) Interactions between α1′ and α3. Residues within the α1′ (Arg105) and α3 (Glu156) helices are colored cyan. The residues in the loop regions of these helices (Ser109, Asn160 and Ile162) are colored gray.

**Figure 4:**
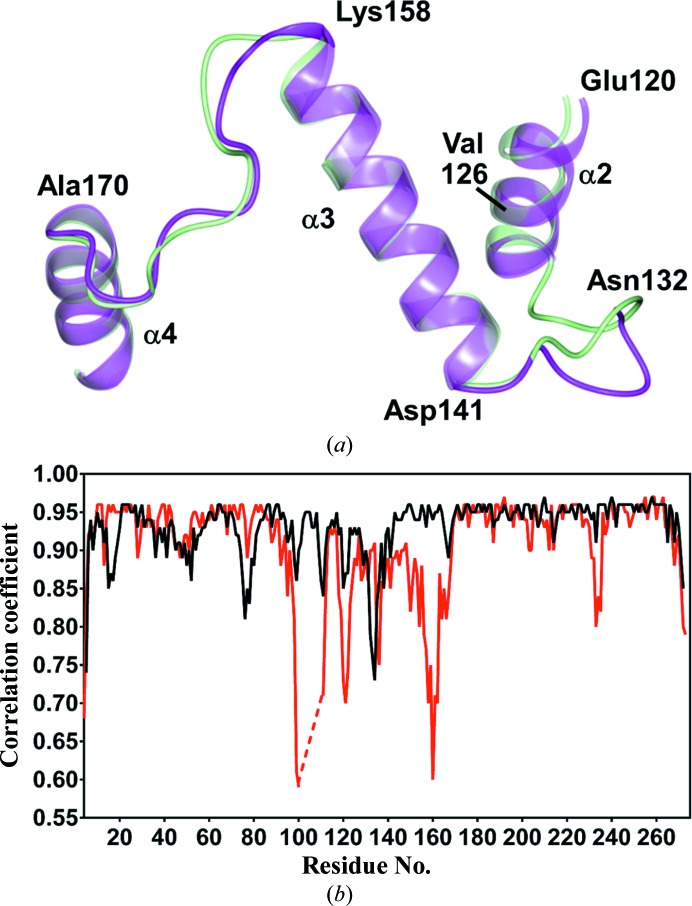
(*a*) Comparison of the regions connecting helices α2 and α3 and helices α3 and α4 in ToxT (PDB entry 4mlo; magenta) with the previously determined structure (PDB entry 3gbg; Lowden *et al.*, 2010 ▸; green). (*b*) Comparison of map correlation coefficients (2*F*_o_ − *F*_c_) for the ToxT structures PDB entry 4mlo (black) and PDB entry 3gbg (red). The dashed line represents disordered residues in PDB entry 3gbg.

The overall structure is similar to PDB entry 3gbg reported by Lowden *et al.* (2010 ▸), with an r.m.s.d. between C^α^ atoms of 1.00 Å (Lys5–Gly272) as determined using the *Secondary Structure Matching* (*SSM*; Krissinel & Henrick, 2004 ▸) algorithm with *SUPERPOSE* *via* the *CCP*4 interface (Winn *et al.*, 2011 ▸). However, there are also differences between the two structures, as shown in the per-residue r.m.s.d. plot in Fig. 2 ▸(*a*) and the superimposed structures in Fig. 2 ▸(*b*). Specifically, the region between α1 and β9, which was disordered in PDB entry 3gbg (Lowden *et al.*, 2010 ▸) from Asp101 to Glu110, could be fully traced in the current structure (Fig. 3 ▸ *a*). In this region, helix α1 spans Ser87–Ile98 and contains a kink at Leu94. This is followed by a 3_10_-helix spanning Leu99–Asp101 that continues into a shorter helix from Leu102 to Leu107 (α1′). Tyr108–Asp113 form a connecting loop between α1′ and β9. This region appears to be stabilized by residue Glu156, in helix α3, through a salt bridge with residue Arg105. Additionally, this region is stabilized by Asn160 and Ile162, from a loop connecting α3 and α4, through hydrogen-bonding interactions with Ser109 of the loop region (Fig. 3 ▸ *b*). The loop region connecting helices α3 and α4 also shows conformational differences relative to PDB entry 3gbg (Lowden *et al.*, 2010 ▸), as depicted in Fig. 4 ▸(*a*), potentially owing to interactions between residues in the previously disordered region and residues in helix α3. Interestingly, the ToxT region between α1 and β9 (residues Asp101–Glu110) is folded over a loop that is located sequentially after it: the loop that connects helices α3 and α4, spanning residues Lys158–Ala170. A very similar arrangement can be observed in the structure of the regulatory domain of ExsA, where the loop connecting α1 and β9 folds over helix α4 (PDB entry 4zua; Shrestha *et al.*, 2015 ▸). ExsA is an AraC-family transcriptional activator that regulates type 3 secretion-system genes in *Pseudomonas aeruginosa* (Shrestha *et al.*, 2015 ▸; Urbanowski *et al.*, 2005 ▸).

Our observation that Arg105 forms a salt bridge with Glu156 may help to explain the prior finding that alanine substitutions of residues Met103, Arg105 and Asn106, within the region that was disordered in PDB entry 3gbg (Lowden *et al.*, 2010 ▸), had a threefold to fourfold elevated activity at the *ctxA* promotor (Childers *et al.*, 2007 ▸). Our structure suggests the possibility that Arg105 holds Glu156 in a position that somewhat attenuates ToxT activity. Glu156 is located in helix α3, which is likely to be involved in dimerization to facilitate transcriptional activation (Lowden *et al.*, 2010 ▸). Thus, Arg105 may maintain the activity of ToxT at its wild-type level by supressing dimerization somewhat (relative to the Arg105Ala substitution). However, other than their potential effects on Arg105, the structure does not provide potential explanations for how alanine substitutions at residues Met103 or Asn106 also increase ToxT activity.

Further analysis was conducted to gauge the quality of fit of the models to the electron density. Analysis of the map–model correlation coefficients *via* *PHENIX* revealed several regions in PDB entry 3gbg that display low correlation to the 2*F*_o_ − *F*_c_ map, including the α3–α4 (Lys158–Ala170) loop (interdomain linker), as shown in Fig. 4 ▸(*b*). Although the Lys158–Ala170 loop region was modeled in PDB entry 3gbg, it was poorly defined, making it difficult to discern the exact positions of the residues in this region. By contrast, the electron density in the current structure was clearly traceable in this region, which is reflected by the high correlation coefficient. It should be noted that none of the residues in this loop form hydrogen-bond contacts with symmetry-related molecules, which suggests that crystal packing was not a factor in the conformational differences relative to PDB entry 3gbg. Additional differences between the two structures were observed in the loop connecting helices α2 and α3 (Asn132–Asp141) and in part of helix α2 (Glu120–Val126) (Fig. 4 ▸ *b*). Gly133 in PDB entry 3gbg was ordered, and was stabilized by Lys4 through hydrogen-bonding interaction. However, both Gly133 and Lys4 were missing from the current structure. It is likely that the slight conformational change in the connecting-loop region (Asn132–Asp141) disrupted the hydrogen-bonding inter­action between Gly133 and Lys4, causing both residues to become flexible and untraceable in the current structure. An alanine substitution of Gly133 had wild-type activity at the *ctxA* promoter (Childers *et al.*, 2007 ▸), suggesting that this residue may not play a key role in the activity of ToxT.

Virstatin, a small-molecule inhibitor of ToxT identified by Hung *et al.* (2005 ▸), blocks ToxT dimerization and thus its ability to activate transcription of the *tcp* and *ctx* promoters (Shakhnovich *et al.*, 2007 ▸). Shakhnovich *et al.* (2007 ▸) also demonstrated that a ToxT variant, Leu114Pro, is resistant to virstatin and suggested that the Leu114Pro mutation may result in a conformational change in ToxT that allows the protein to dimerize more efficiently (Shakhnovich *et al.*, 2007 ▸). Lowden *et al.* (2010 ▸) suggested that the previously disordered region from Asp101 to Glu110 might be involved in the virstatin resistance of the Leu114Pro variant owing to its proximity; however, there are no obvious interactions between Leu114 and any of the residues in the 101–110 region that would suggest involvement of this region in the mechanism of virstatin resistance of ToxT Leu114Pro.

Overall, the new 1.65 Å resolution crystal structure of ToxT (PDB entry 4mlo) reveals the structure of the previously unresolved region (residues 101–110), including the presence of a previously unidentified helix (α1′), as well as interactions between the residue 101–110 region and surrounding residues. This region is of importance as substitutions have been shown to effect activation of the *ctxA* promotor (Childers *et al.*, 2007 ▸). There are several additional structural differences between the previously reported structure (PDB entry 3gbg; Lowden *et al.*, 2010 ▸) and the new structure (PDB entry 4mlo). Overall, the new structure provides more complete, detailed and higher quality structural information for ToxT than the previously determined ToxT structure.

## Supplementary Material

PDB reference: ToxT from *Vibrio cholerae*, *P*2_1_ form, 4mlo

## Figures and Tables

**Table 1:** Data-collection and refinement statistics for the ToxT structure Values in parentheses are for the highest resolution shell.

Data collection	
Unit-cell parameters (Å, °)	*a* = 47.34, *b* = 39.41, *c* = 80.24, β = 97.94
Space group	*P*2_1_
Resolution (Å)	39.73–1.65 (1.68–1.65)
Wavelength (Å)	1.0000
Temperature (K)	100
Observed reflections	117532
Unique reflections	35493
〈*I*/σ(*I*)〉	10.3 (1.9)
Completeness (%)	99.6 (99.8)
Multiplicity	3.3 (3.4)
*R*_merge_ † (%)	8.1 (68.0)
*R*_meas_ ‡ (%)	9.7 (82.6)
*R*_p.i.m._ ‡ (%)	5.2 (43.1)
CC_1/2_ §	0.997 (0.714)
Refinement	
Resolution (Å)	39.74–1.65
Reflections (working/test)	33700/1777
*R* factor/*R*_free_ ¶ (%)	16.8/19.4
No. of atoms	
Protein	2181
Chloride	3
PAM	18
Water	179
Model quality	
R.m.s. deviations	
Bond lengths (Å)	0.009
Bond angles (°)	0.947
Average *B* factor (Å^2^)	
All atoms	25.9
Protein	25.5
Chloride	16.6
PAM	28.1
Water	30.7
Coordinate error (maximum likelihood) (Å)	0.17
Ramachandran plot	
Most favored (%)	99.6
Additionally allowed (%)	0.4

† *R*_merge_ = 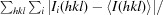 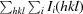, where *I_i_*(*hkl*) is the intensity measured for the *i*th reflection and 〈*I*(*hkl*)〉 is the average intensity of all reflections with indices *hkl*.

‡ *R*_meas_ is the redundancy-independent (multiplicity-weighted) *R*_merge_ (Evans, 2006 ▸, 2012 ▸). *R*_p.i.m._ is the precision-indicating (multiplicity-weighted) *R*_merge_ (Diederichs & Karplus, 1997 ▸; Weiss, 2001 ▸).

§ CC_1/2_ is the correlation coefficient of the mean intensities between two random half-sets of data (Karplus & Diederichs, 2012 ▸; Evans, 2012 ▸).

¶ *R* factor = 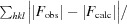 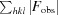; *R*_free_ is calculated in an identical manner using a randomly selected 5% of the reflections, which were not included in the refinement.

## References

[bb1] Adams, P. D. *et al.* (2010). *Acta Cryst.* D**66**, 213–221.

[bb2] Brown, R. C. & Taylor, R. K. (1995). *Mol. Microbiol.* **16**, 425–439. 10.1111/j.1365-2958.1995.tb02408.x 7565104

[bb3] Champion, G. A., Neely, M. N., Brennan, M. A. & DiRita, V. J. (1997). *Mol. Microbiol.* **23**, 323–331. 10.1046/j.1365-2958.1997.2191585.x 9044266

[bb4] Chatterjee, A., Dutta, P. K. & Chowdhury, R. (2007). *Infect. Immun.* **75**, 1946–1953. 10.1128/IAI.01435-06 PMC1865667 17261615

[bb5] Chen, V. B., Arendall, W. B., Headd, J. J., Keedy, D. A., Immormino, R. M., Kapral, G. J., Murray, L. W., Richardson, J. S. & Richardson, D. C. (2010). *Acta Cryst.* D**66**, 12–21. 10.1107/S0907444909042073 PMC2803126 20057044

[bb6] Childers, B. M., Weber, G. G., Prouty, M. G., Castaneda, M. M., Peng, F. & Klose, K. E. (2007). *J. Mol. Biol.* **367**, 1413–1430. 10.1016/j.jmb.2007.01.061 17320105

[bb7] Diederichs, K. & Karplus, P. A. (1997). *Nature Struct. Biol.* **4**, 269–275. 10.1038/nsb0497-269 9095194

[bb8] DiRita, V. J., Parsot, C., Jander, G. & Mekalanos, J. J. (1991). *Proc. Natl Acad. Sci. USA*, **88**, 5403–5407. 10.1073/pnas.88.12.5403 PMC51881 2052618

[bb9] Egan, S. M. (2002). *J. Bacteriol.* **184**, 5529–5532. 10.1128/JB.184.20.5529-5532.2002 PMC139625 12270809

[bb10] Emsley, P., Lohkamp, B., Scott, W. G. & Cowtan, K. (2010). *Acta Cryst.* D**66**, 486–501. 10.1107/S0907444910007493 PMC2852313 20383002

[bb11] Evans, P. (2006). *Acta Cryst.* D**62**, 72–82. 10.1107/S0907444905036693 16369096

[bb12] Evans, P. R. & Murshudov, G. N. (2013). *Acta Cryst.* D**69**, 1204–1214. 10.1107/S0907444913000061 PMC3689523 23793146

[bb13] Evans, P. (2012). *Science*, **336**, 986–987. 10.1126/science.1222162 22628641

[bb14] Gallegos, M.-T., Michán, C. & Ramos, J. L. (1993). *Nucleic Acids Res.* **21**, 807–810. 10.1093/nar/21.4.807 PMC309210 8451183

[bb15] Gallegos, M.-T., Schleif, R., Bairoch, A., Hofmann, K. & Ramos, J. L. (1997). *Microbiol. Mol. Biol. Rev.* **61**, 393–410. 10.1128/mmbr.61.4.393-410.1997 PMC232617 9409145

[bb16] Higgins, D. E., Nazareno, E. & DiRita, V. J. (1992). *J. Bacteriol.* **174**, 6974–6980. 10.1128/jb.174.21.6974-6980.1992 PMC207377 1400247

[bb17] Hung, D. T., Shakhnovich, E. A., Pierson, E. & Mekalanos, J. J. (2005). *Science*, **310**, 670–674. 10.1126/science.1116739 16223984

[bb18] Ibarra, J. A., Pérez-Rueda, E., Segovia, L. & Puente, J. L. (2008). *Genetica*, **133**, 65–76. 10.1007/s10709-007-9185-y 17712603

[bb19] Kabsch, W. (1988). *J. Appl. Cryst.* **21**, 67–72.

[bb20] Karplus, P. A. & Diederichs, K. (2012). *Science*, **336**, 1030–1033. 10.1126/science.1218231 PMC3457925 22628654

[bb21] Krissinel, E. & Henrick, K. (2004). *Acta Cryst.* D**60**, 2256–2268. 10.1107/S0907444904026460 15572779

[bb22] Lowden, M. J., Skorupski, K., Pellegrini, M., Chiorazzo, M. G., Taylor, R. K. & Kull, F. J. (2010). *Proc. Natl Acad. Sci. USA*, **107**, 2860–2865. 10.1073/pnas.0915021107 PMC2840316 20133655

[bb23] Matthews, B. W. (1968). *J. Mol. Biol.* **33**, 491–497. 10.1016/0022-2836(68)90205-2 5700707

[bb24] McCoy, A. J., Grosse-Kunstleve, R. W., Adams, P. D., Winn, M. D., Storoni, L. C. & Read, R. J. (2007). *J. Appl. Cryst.* **40**, 658–674. 10.1107/S0021889807021206 PMC2483472 19461840

[bb26] McNicholas, S., Potterton, E., Wilson, K. S. & Noble, M. E. M. (2004). *Acta Cryst.* D**67**, 386–394. 10.1107/S0907444911007281 PMC3069754 21460457

[bb25] Painter, J. & Merritt, E. A. (2006). *Acta Cryst.* D**62**, 439–450. 10.1107/S0907444906005270 16552146

[bb40] Ramos, J. L., Rojo, F., Zhou, L. & Timmis, K. N. (1990). *Nucleic Acids Res.* **18**, 2149–2152. 10.1093/nar/18.8.2149 PMC330695 2186376

[bb27] Schuhmacher, D. A. & Klose, K. E. (1999). *J. Bacteriol.* **181**, 1508–1514. 10.1128/jb.181.5.1508-1514.1999 PMC93540 10049382

[bb28] Shakhnovich, E. A., Hung, D. T., Pierson, E., Lee, K. & Mekalanos, J. J. (2007). *Proc. Natl Acad. Sci. USA*, **104**, 2372–2377. 10.1073/pnas.0611643104 PMC1892951 17283330

[bb29] Shrestha, M., Xiao, Y., Robinson, H. & Schubot, F. D. (2015). *PLoS One*, **10**, e0136533. 10.1371/journal.pone.0136533 PMC4552939 26317977

[bb30] Studier, F. W. (2005). *Protein Expr. Purif.* **41**, 207–234. 10.1016/j.pep.2005.01.016 15915565

[bb31] Tobes, R. & Ramos, J. L. (2002). *Nucleic Acids Res.* **30**, 318–321. 10.1093/nar/30.1.318 PMC99111 11752325

[bb32] Urbanowski, M. L., Lykken, G. L. & Yahr, T. L. (2005). *Proc. Natl Acad. Sci. USA*, **102**, 9930–9935. 10.1073/pnas.0504405102 PMC1175016 15985546

[bb33] Weiss, M. S. (2001). *J. Appl. Cryst.* **34**, 130–135.

[bb34] Winn, M. D. *et al.* (2011). *Acta Cryst.* D**67**, 235–242.

[bb35] Winn, M. D., Isupov, M. N. & Murshudov, G. N. (2001). *Acta Cryst.* D**57**, 122–133. 10.1107/s0907444900014736 11134934

[bb36] Yu, R. R. & DiRita, V. J. (1999). *J. Bacteriol.* **181**, 2584–2592. 10.1128/jb.181.8.2584-2592.1999 PMC93687 10198025

